# Booster Vaccination against Yellow Fever in Gambian children-(BoVY) -a Phase 3 clinical trial to establish safety and immunogenicity of repeated YF vaccination in healthy Gambian children of different ages

**DOI:** 10.12688/wellcomeopenres.23138.1

**Published:** 2024-12-24

**Authors:** Beate Kampmann, Caitlin Pley, Julia Strandmark, Mam Nabou Leigh, Peter Ndow, Ed Clarke, Elishia Roberts, Amadou Faal, David Jeffries, Ebrima Kanteh

**Affiliations:** 1 Centre for Global Health, Charité Universitätsmedizin Berlin, Berlin, 10117, Germany; 2 Vaccines & Immunity Theme, MRC Unit The Gambia at the London School of Hygiene and Tropical Medicine, Fajara, The Gambia; 3 Clinical Research Department, London School of Hygiene & Tropical Medicine, London, England, UK

**Keywords:** Yellow Fever, vaccine, booster, WHO guidelines, Clinical trial, Children, The Gambia, Global Health

## Abstract

**Background:**

Yellow fever (YF) is a mosquito-borne and recently re-emerging viral haemorrhagic fever endemic to sub-Saharan Africa and South America. A highly effective vaccine against YF is licensed and recommended as part of routine childhood immunisation as a single dose at 9 months. Recent observational data demonstrate waning immunity following single primary vaccination and suggest that children in endemic areas may require booster vaccination.

**Methods:**

This open-label, non-randomised clinical vaccine trial (ClinicalTrials.gov, NCT05332197, registered on 31 March 2022, URL: https://clinicaltrials.gov/study/NCT05332197) will assess the safety and immunogenicity of a booster dose of the licensed 17D YF vaccine in Gambian children. The trial will recruit 750 children in three cohorts of different ages (250 each). All children were vaccinated with the 17D YF vaccine at 9–10 months of age as part of clinical trials run by the Medical Research Council (MRC) Unit The Gambia, and are thus well-characterised, including basic clinical, anthropometric, and post-primary immunogenicity data. The children will receive booster doses at 15 months, 4 years, or 8.5 years. Serum samples will be taken before and 28 days after the booster, with additional sampling for exploratory endpoints in subgroups. Adverse events are solicited for the first three days following vaccination and recorded throughout the study period. The primary objective of the trial is to describe the safety and immunogenicity of the booster in the different age cohorts. Secondary objectives are to characterise the rate of sero-reversion (change from seropositive to seronegative) over a period of 9 months to 8 years following single primary vaccination and to profile the immune response to the booster to explore underlying mechanisms for the longevity of vaccine-induced antibody.

**Discussion:**

The results of this trial are likely to directly impact WHO recommendations on whether booster vaccination is required for children in endemic areas, and if so, the optimal timing of such a booster.

## Plain Language Summary

**Why is it important?:**

Yellow fever is a potentially deadly disease that is found across sub-Saharan Africa and South America. Fortunately, an effective vaccine exists and is used in affected countries to protect children and adults against the disease. Current World Health Organization (WHO) guidelines recommend that a single vaccination is sufficient to provide life-long protection. However, recent data show that many children who are vaccinated when they are very young (especially under the age of 12 months) have decreasing immune responses over time, meaning they may become vulnerable to yellow fever again as they get older.

**Research overview:**

This study aims to understand whether children who were vaccinated against yellow fever when they were very young require a booster dose of the vaccine, and if so, at which age this booster dose should be given. This study was conducted by the Medical Research Council unit in The Gambia, in West Africa, from 2022 to 2024. Children who were vaccinated against yellow fever at 9–10 months of age as part of the normal childhood vaccination schedule were recruited with parental consent. Three different cohorts of children were included and given yellow fever boosters at ages 15 months, 4 years, or 8.5 years. The researchers tested the children’s antibody levels before and after the booster to understand how the children of different ages responded to the booster. The researchers could also compare their results against the children’s known antibody levels after their first vaccination to understand how these had changed over time.

**Key takeaway:**

This research will help clinicians, scientists and policymakers better understand the decline of immune responses after primary yellow fever vaccination in early childhood and evaluate the need and optimal timing for a booster vaccine.

## Abbreviations

**AE**     Adverse Event

**AEFI**    Adverse Event Following Immunization

**CRF**    Case Report Form

**DMP**    Data Management Plan

**DSMB**    Data Safety and Monitoring Board

**ELISpot **   Enzyme-linked Immunosorbent Spot

**GCP**    Good Clinical Practice

**GMT**    Geometric Mean Titre

**ICH**    International Conference on Harmonization

**IEC**    Independent Ethics Committee

**MCA**    Medicines Control Agency, The Gambia

**MCV**    Measles-containing vaccine

**MRCG**   Medical Research Council Unit The Gambia at the London School of Hygiene & Tropical Medicine

**PBMC**   Peripheral blood mononuclear cell

**PI**   Principal Investigator

**PD**   Protocol deviation

**PRNT**   Plaque Reduction Neutralization Test

**RCD**   Reverse cumulative distribution

**SAE**   Serious Adverse Event

**SAGE**   Strategic Advisory Group of Experts

**SUSAR**   Suspected Unexpected Serious Adverse Reaction

**WHO**   World Health Organization

**YF**   Yellow Fever

## Introduction

### Background

Yellow fever (YF) virus is a mosquito-borne flavivirus found in Sub-Saharan Africa and tropical South America. The virus causes YF, a viral haemorrhagic fever, which can be prevented by a highly effective live-attenuated vaccine, however, it is now considered a re-emerging disease due to the increased numbers of cases in the last 30 years ^1^. Across Africa, 13 countries in West, Central and East Africa reported yellow fever cases in 2023 and 2024, with a case fatality rate of 11% ^2^. The median age of these cases was 25 years, with over two thirds of cases (69%) over the age of 15 years, suggesting a potential contribution of waning immunity to increased disease susceptibility ^2^.

In most endemic countries across sub-Saharan Africa and South America, the YF vaccine is administered alongside other Expanded Programme on Immunization (EPI) vaccines, including the first measles containing vaccine (MCV1), at nine to 12 months of age. Several recent publications from Africa and Brazil have raised doubts that a single dose of YF vaccine provides sufficiently long-lasting immunity. In a cohort of children vaccinated at around nine months of age in Ghana, seropositivity, measured by microneutralization assay, had fallen from an already low 73% at 28 days following primary vaccination to 28% at 2.3 years ^3^. This figure had increased to 43% by six years, perhaps as a result of natural exposure or unrecorded re-vaccination. Our own data from samples collected in The Gambia and Mali 6 years post primary YF vaccination administered via the routine EPI program showed that 22.2% of a cohort of 467 children had undetectable antibody concentrations, with another 7.5% revealing concentrations below the threshold of seropositivity of 0.5 IU/mL, confirming a substantial long-term decline in immunity ^4^. Cross-sectional studies in Brazil, including nine-month to 12-year olds, have similarly described a progressive decrease in the seropositivity rates from 87% within the first six-months after vaccination to 42% at six to eight years ^5^. A recent systematic review summarised the findings of 36 studies on over 17,000 participants aged 6 months to 85 years from 20 countries across the world, including Sub-Saharan Africa ^6^. In children vaccinated under the age of 2 years, the pooled seroprotection rate in a meta-analysis using a random effects model was 52% at 5 years following primary vaccination ^6^. In addition to waning immunity over time following primary vaccination, the co-administration of the YF vaccine with the first dose of MCV1 in many EPI programmes may contribute to a lack of protection, as a number of studies have shown that MCV1 co-administration interferes with the generation of YF immunity ^7, 8^. Concomitant infections and immunological interference from persistent maternal antibody may also contribute ^9, 10^.

It remains difficult to directly link the declining titres to actual occurrence of new cases of YF disease, as detailed immunisation history and in particular PRN titres are generally not available in observational studies of new cases, and no efficacy study has ever been performed. It is generally accepted, however, that high titres of YF antibodies protect against disease ^11^. Neutralising antibodies, in particular, are considered the primary mode of protection following YF vaccination and constitute the accepted correlate of protection for the disease ^11^. The low residual titres in young children are therefore a legitimate cause for concern ^12^. Our understanding of the role of cell-mediated immunity following YF vaccination is also advancing; although T-cell responses are believed to contribute to protection against disease, YF’s short incubation period underscores the importance of memory B cells and a rapid antibody response ^11^.

### Rationale

Despite the vaccine being very successful at decreasing disease risk, YF is considered a re-emerging disease due to the increased numbers of cases in the last 30 years. Until 2014, the vaccine was recommended to be administered with boosters every 10 years, but in 2014 the World Health Organization recommended removal of booster doses for all except special populations ^13^. This recommendation has been questioned and there have been reports of waning antibody titres in adults over time, and more recently also reports in paediatric populations. The scarcity of long-term studies on neutralising antibodies elicited in vaccinated infants has been identified as a knowledge gap with regard to the need for booster doses of the yellow fever vaccine ^11, 14^. Based on these emerging data, we need to establish whether infants who received a primary dose of a yellow fever vaccine at nine months of age, in line with EPI recommendations, are indeed likely to require a booster dose of the vaccine in order to ensure long-term protection.

We have a unique opportunity to address these questions of timing and immunogenicity which are of public health importance. The proposed trial leverages three well-characterized cohorts of infants in The Gambia, all of whom were vaccinated with the Institut Pasteur de Dakar 17D YF vaccine at nine to 10 months of age within clinical trials run at the MRC Unit The Gambia at the London School of Hygiene and Tropical Medicine (MRCG). Details of the vaccine lots used and other metadata, including anthropometric measurements and other health indices related to the infants at the time of vaccination, are available. These trials were all conducted with ethical approval at the MRC Unit in The Gambia and already have consent for future use of data and stored samples in the original informed consent documentation. For most children in these cohorts, the initial serological responses to the vaccine are already documented from assays previously conducted at the Robert Koch Institute in Berlin, and for others, serum samples from 28 days following the primary immunization remain in secure -80°C storage. At the time of the proposed study, the cohorts will range in age from between 15 and 18 months, to between eight and nine years. These cohorts therefore provide a unique opportunity not only to examine antibody decline over this time period, but also to determine the optimal timing of a booster dose following infant priming. In addition, they allow for comprehensive immune-profiling of innate and cellular responses to YF vaccination in the different age groups to generate new insights into determinants of long-term immunity. Such data have never been generated in paediatric populations.

## Study protocol (V2.0, July 2022)

This clinical trial protocol was registered on ClinicalTrials.gov on 31 March 2022 under registry number NCT05332197 and is available from https://clinicaltrials.gov/study/NCT05332197 ^15^.

### Study objectives

The main objective of this trial is to assess if a yellow fever (YF) booster dose is required for children who received a single dose of YF vaccination in infancy and if so, at what age this booster dose might be most effective to administer.

Primary:

- To describe the safety and immunogenicity of a booster dose of a licensed YF vaccine administered to three different age cohorts of children, following a documented primary dose of a yellow fever vaccine administered at nine months of age.

Secondary:

- To characterise the rate of YF PRNT seroreversion (seropositive to seronegative) over a period of 9 months to 8 years following a single primary dose of yellow fever vaccine administered to Gambian infants at nine months of age.
- To profile the immune response to the booster dose of YF vaccine in order to explore underlying mechanisms for longevity of vaccine-induced antibody.

### Potential risks and benefits

The risks are low as this is an already licensed vaccine and boosters have previously been recommended for adults with no safety concerns ^16^. The benefit is expected to be added protection against YF infection based on an increase in PRNT titres, the established correlate of protection. Participants may also benefit from being assessed by a study clinician at the time of enrolment. Should any health complaints be identified during this screening, initial treatment will be provided by the study team and the participant will be referred onwards for ongoing care, should this be necessary, at a government health facility.

### Study endpoints

Primary:

The primary endpoint of this study is the PRNT _50_ titres post booster vaccination according to intervals between primary Yellow Fever vaccination and the booster dose.

Secondary:

The secondary endpoint is the rate of YF PRNT seroreversion (seropositive to seronegative) over a period of 9 months to 8 years following a single primary dose of YF vaccine administered to Gambian infants at nine months age.

Exploratory (Sub-study only):

- Frequency of activated T cells, following re-stimulation with YF, in children of different ages following a booster dose of YF vaccination
- Levels of YF specific memory B cells in children of different ages following a booster dose of YF vaccination
- Possible impact of early innate responses, as measured by RNASeq, on antibody, B and T cell responses

### Study design

This is a Phase 3 clinical trial to establish safety and immunogenicity of a booster dose of licensed YF vaccine in children of different age groups, following primary immunization with the same vaccine at 9–10 months of age.

**Figure 1.**
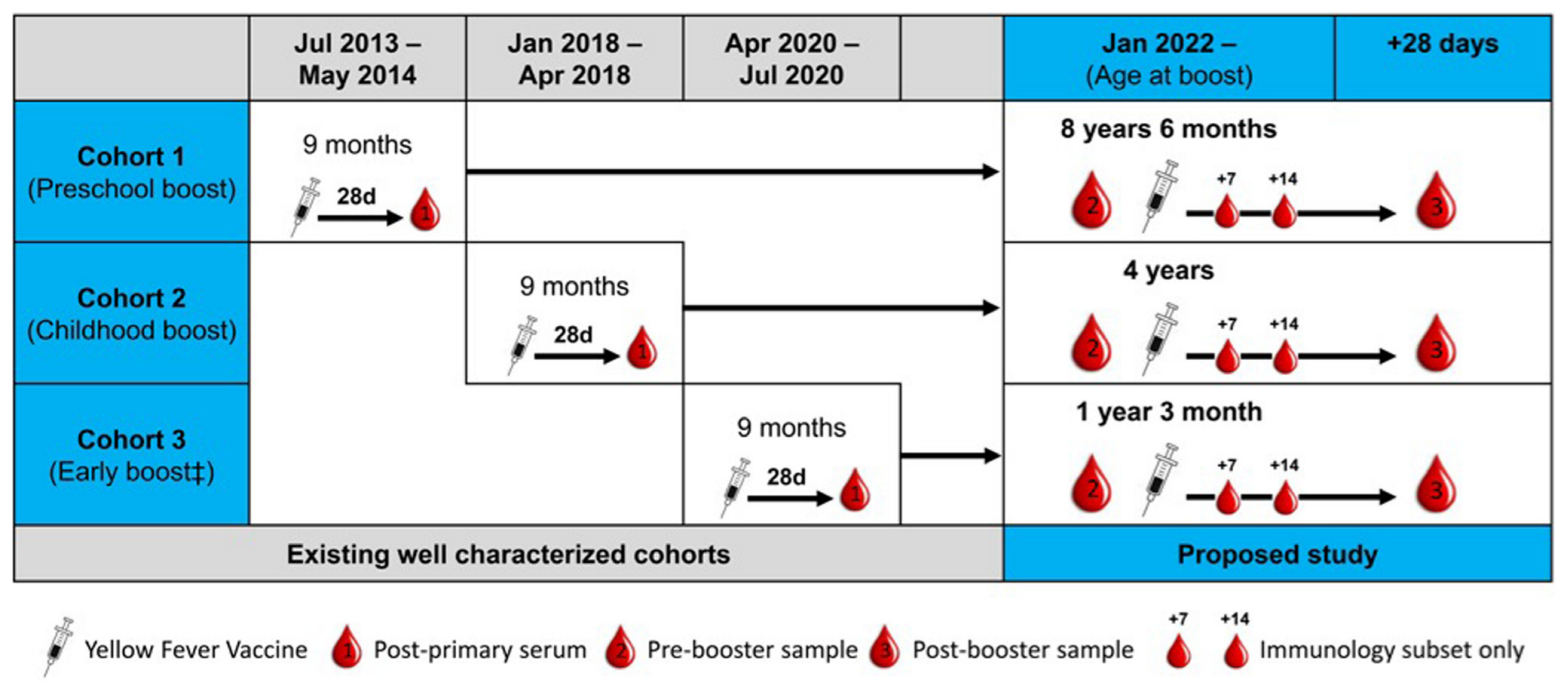
Study design.

The proposed study leverages three well-characterized cohorts of infants, all of whom were vaccinated with the Institut Pasteur de Dakar 17D YF vaccine at 9–10 months of age within clinical trials run by the Vaccines & Immunity Theme in the MRC Unit The Gambia at the London School of Hygiene and Tropical Medicine (MRCG) over the last few years ( Figure 1). The databases of the previous trials, the YF PRNT _50_ results from the primary immunisation and the stored samples are immediately available to the investigators. Consent for sample storage and future work on remaining samples was obtained at the time of initial recruitment.

This trial will be conducted at one site: Fajikunda Health Centre in Fajara, The Gambia. Children previously enrolled in each of the three historic vaccine trials (summarised in Table 1) will be recalled and invited for participation in the booster study. We will enrol until we have re-recruited 250 children per cohort. A roughly equal number of male and female participants will be enrolled in each cohort, but no absolute percentage figures will be set. Following individual sensitization and informed consent, basic clinical and anthropometric data will be collected, and children will have a pre-booster blood sample (5–10ml depending on age) taken for YF PRNT _50_ ( Figure 1). All children will then receive a booster dose of a licensed 17D YF vaccine in use in The Gambia. All vaccines will be administered by the MRCG team. Local and systemic reactogenicity data will be solicited for 3 days. All children will then be invited back at day 28 (+/-7-day window) for a second blood sample (5–10 ml depending on age) and PBMC will be isolated and stored at the same timepoint ( Figure 1). If, based on initial laboratory work, it is determined that a smaller number of PBMC will be required at this timepoint, only serum will be collected for post-booster YF PRNT _50_. A smaller cohort in each age group will be enrolled in the sub-study described below.

***Sub-studies***

In order to conduct systems biology investigations, which will provide novel insights into YF immune responses in children, subgroups of 30 children in each cohort will also be consented for a further sample (max. 5 ml) at either Day +7 for RNASeq or Day +14 for analysis of T cell responses ( Figure 1). The aim is to enrol the first 30 eligible children per cohort in the sub-studies, following consent provided on consent form version 1.0. Any child in these cohorts will have a maximum of 3 blood samples (up to 5 ml in the youngest age group, up to 10 ml/timepoint in the older age groups.)

### Laboratory investigations

***Humoral immune responses***

Serum collected pre and post YF vaccination will be separated from clotted whole blood samples, aliquoted and frozen at below -70°C prior to analysis at either the Institut Pasteur in Dakar or at the Robert Koch Institute in Berlin. This assay is not available in The Gambia. Post-primary serum from the primary studies is already available and safely stored for all participants and will also be analysed where data are not already available. Serum will be shipped using an IATA qualified shipping agent. A back-up sample will be retained at MRCG. YF neutralizing antibody titres will be determined by a validated PRNT assay and results provided to MRCG for subsequent analysis. The PRNT assay is conducted on a 96-well plate. The serum samples will first be inactivated at 60° Celsius for 20 minutes and then serial two-fold dilutions (from 1:10 to 1:20480) will be made. 30μL of the diluted serum samples will be added to the wells with 30μL of virus stock (containing 10 ^3^ pfu/ml). The Institut Pasteur Yellow Fever 17D vaccine is used as the challenge strain for this PRNT and is titrated using serial dilution in a 24-well plate and through the observation of cytopathic effect in Porcine Stable (PS) kidney cells, measured as plaque-forming units (pfu). 30μL of cell culture medium will also be added to each well. Cell control, non-diluted virus and reference positive serum (National Institute for Biologicals Standards and Control) will be used as controls. The mixtures will then be incubated at 37° Celsius for 1 hour, after which another 6x10 ^4^ PS cells will be added to each well. After 3–4 hours incubation at 37° Celsius, an overlay medium (0.6% carboxymethyl-cellulose in Fetal Bovine Serum (FBS)) will be added to each well and plates will be incubated at 37° Celsius for 3–4 days. Cytopathic effect will be observed using a standard microscope at x40 magnification. Subsequently, the medium will be removed, and the cells washed twice before 150μL of amido black (Sigma Aldrich) will be added to each well to stain and fix the cells to count the plaque-forming units. The PRNT90 and -50 antibody titres are defined as the last dilution that reduces the plaque forming units by 90% or 50% respectively when compared to the control. This YF PRNT assay has been optimised and validated ^17^.

***Cellular immune responses***

It is well established that both CD4 and CD8 T cell responses are elicited by YF vaccination ^18^, although their role in vaccine efficacy is not fully clarified. Recently published data in adults from a non-endemic setting have shown that both antibody and CD8 T cell responses might be impaired by a YF booster vaccine, compared to responses to the primary vaccine ^18^, however, there are no data in children.

This study provides an excellent opportunity to further understand the impact of a booster dose on cellular immunity and its relationship, if any, to neutralising YF antibody. In addition to antibody analyses prior and post YF booster vaccination and storage of PBMC, subgroups of children (n=30) in each study cohort will therefore be sampled either at D+7 for RNA sequencing or D+14 to study T cell adaptive responses.

***PBMC separation and storage***

PBMC will be isolated and stored. If, based on initial laboratory work, it is determined that a smaller number of PBMC will be required at this timepoint, serum will be collected alone. To separate the PBMCs, 3–5ml of blood will be collected in heparin tubes (BD Biosciences, CA, USA). The blood will be diluted using RPMI-1640, containing 100U/ml Penicillin and 100 µg/ml Streptomycin, together with 3% heat inactivated FBS (HI-FBS). This diluted blood will be layered over Lymphoprep at a ratio of 2:1 and then spun at 2400 rpm (1037g) for 30 minutes. The separated PBMCs will then be washed twice and resuspended in complete medium with 1% L-glutamine and 10% FBS. Cell counting will be performed using a Luna II automated cell counter (Logos Biosystems, South Korea). The cells will then be frozen in 10% DMSO in HI-FBS at -80°Celsius. After 48 hours, the cells will be moved to liquid nitrogen for further storage.

***Memory B cell assay***

To relate the serological responses to the B cell memory pool, memory B cell ELISpot assays will be carried out on fresh or banked PBMC from the V1 (baseline) and V2 (D+28) blood samples for all children where possible ^19^. Frozen PBMCs will be thawed and pre-incubated for 6 days with polyclonal antigen (stimulated PBMCs) or complete medium (unstimulated PBMCs). Stimulated and unstimulated cells will then be added to ELISpot plates, pre-coated with either 17D (to measure YF antigen-specific cells) or with anti-human IgG antibodies (to measure total IgG secretion). To allow direct comparison, PBMCs from the same subject, derived from visit 1 (day 0) and 2 (day 28) will be run on the same plate. Following incubation, biotinylated anti-human IgG will be added to detect antigen-specific and non-specific memory B cells. The addition of an alkaline phosphatase substrate creates insoluble coloured spots on the wells, which are enumerated using an automated ELISpot reader, with each spot corresponding to a single memory B cell.

***Flow cytometry and ExVivo staining for surface activation markers***

1x10 ^6^ thawed PBMCs will first be washed in FACS buffer, which consists of PBS supplemented with 0.5mM EDTA and 3% HI-FBS (all Sigma-Aldrich, UK). The PBMCs will then be stained with the viability marker LIVE/DEAD™ (Invitrogen™) followed by surface staining using conjugated antibodies against CD3_V500, CD4_V450, CD8_FITC, CD38_APC R700 and HLA-DR_PE (all BD Bioscience). The cells will then be run on a BD Symphony™ Flow Cytometer and analysed using DB FACSDiva™ software ^20^ (open access alternative ^21^). Visits from the same participant will be run on the same plate and cohort samples will be distributed equally across plates to minimize batch effects. UltraComp eBeads™ (Invitrogen) will be used as controls. FCS files will be analysed using FlowJo ^22^ (TreeStar, USA). A template gating strategy, based on fluorescent minus one samples, will be used to analyse all FCS files.

***PBMC Restimulation and detection of activation markers***

1x10 ^6^ thawed PBMCs will be stimulated for 18 hours with 5024 IU 17D antigen at 37°Celsius and under 5% CO _2_. 1µg/ml SEB and R10 will be used as positive and negative controls, respectively. After the 18 hours, the cells will be stained with viability dye and the detection of surface markers will be performed, as above, using antibodies against CD3_V500, CD4_V450, and CD8_FITC (all BD Bioscience). After the surface staining, the samples will be fixed in 150µl of Cytofix/Cytoperm solution (BD Cytofix/Cytoperm™) for 15 minutes at 4°Celsius, after which the cells will be washed twice in Perm Wash buffer (BD Perm/Wash™). Intracellular staining will then be done using conjugated antibodies against CD154_APC-Cy7 (BioLegend ^®^), IL_2-APC (BioLegend ^®^), TNFα_AF700 (BD Bioscience), and IFNg_PE (BD Bioscience). Cells will then be incubated in the dark for 60 minutes at 4°Celsius. After a repeated washing in Perm Wash buffer, the cells will then be acquired through flow cytometry as described above. In addition to the template gating strategy, a Boolean gating analysis will be applied to identify T cell subsets expressing combinations of activation markers ^23^.

***RNA sequencing***

On Day 7, whole blood samples (1–2 ml) will be collected into PaxGene tubes and analysed using RNASeq methodology, recently established in the Gambia. It involves extraction of total RNA using the RiboPure RNA purification kit (Ambion ThermoFisher, Waltham, USA), followed by quantification and quality assessment using an Agilent 2100 Bioanalyzer (Santa Clara, USA). Polyadenylated RNA was captured and strand-specific cDNA libraries generated, which were subsequently sequenced using HiSeq 2500 (Illumina, San Diego, USA). This method was recently applied to small volume blood samples of an infant cohort to understand immune ontogeny in unprecedented detail ^24^ and will allow us to investigate whether YF antibody responses to the booster can be predicted by early “omic” signatures, as seen in adults ^25^. To date, this has not been studied in children.

All detailed “omic” and cellular immune studies can be implemented at the MRCG. Where possible, PBMC will be saved to test the hypothesis that the size of remaining B cell memory populations generated by primary YF immunisation will impact on the booster responses observed. The data generated will provide insights into the relationship between early innate responses, T cell activation, B cell profiles and functional antibody. We will use novel systems biology integrative approaches to inter-relate these immune-datasets, as we have recently published ^24^. As has been shown by us and others, systems biology approaches not only permit the observation of a global picture of vaccine-induced innate immune responses but can also be used to predict the magnitude of the subsequent adaptive immune response and uncover new correlates of vaccine efficacy ^25^. This approach has not yet been applied to understanding YF immunity in children and is an exploratory element of the trial.

### Investigational products

The vaccine to be used in this booster study is a licensed 17D YF vaccine, which forms part of the National EPI program of The Gambia, depending on product availability.

***Description of products***

This vaccine contains the Rockefeller17D-substrain 204 strain of Yellow fever virus. The exact potency of each batch is released with the certificate of analysis that accompanies each batch and potency is confirmed at the time of release. This vaccine is one of the YF vaccines previously used by the national EPI program.

***Formulation, packaging and labelling***

This vaccine is presented as a 1–10 dose vial with freeze-dried content which is reconstituted with a diluent, provided together with the vaccine by the manufacturer.

***Product storage and stability***

The vaccine is supplied with a Vaccine Vial Monitor which will be used to determine whether the vaccine is in a condition to be used at any point, including monitoring any temperature deviations.

***Dosage, preparation and administration of investigational products***

Reconstituted vaccines have to be kept in a vaccine carrier at 2–8°Celsius as per WHO and manufacturer requirements. Any remaining reconstituted vaccine not used has to be discarded after 6 hours. The standard dose is 0.5 ml and is administered subcutaneously in the deltoid region using standard vaccination syringes (needle size 25G × 3/4”) with a 45° injection angle.

***Concomitant vaccinations***

Children due additional EPI vaccines during their period of enrolment in the study will have them administered following the day 28 blood sample. These vaccines will either be administered by the study team or the study team will ensure the vaccines are given through the routine EPI clinic at the site. No vaccines will be administered concomitantly with the yellow fever vaccines.

### Selection and withdrawal of participants

***Selection of participants***

The study will recruit 750 healthy children to receive the booster dose of YF vaccine. A roughly equal number of boys and girls will be recruited. We do not expect there to be differences in the results by sex.

Participants will be recruited/ re-approached from the previously enrolled participants in each of the vaccine trials listed in Table 1 below.

**Table 1.** Cohorts to be re-approached as part of the trial.

Dates of 9-month yellow fever vaccination	Age in January 2022	Number of infants	Co-administered vaccines in original study	Serology in primary study	Study reference
Jul 2013 – May 2014	8 years 6 years	~1504	Combinations of IPV and measles and rubella	PRNT _50_	NCT01847872 SCC1327
Feb 2018 – Aug 2018	4 years	~675	Measles and rubella, OPV and PCV	PRNT _50_	NCT03197376 SCC1517
Dec 2020 – May 2022	9 months	~320	Measles and rubella, OPV and PCV	PRNT _50_	NCT03606096 SCC1600

Upon screening, potentially eligible children will continue until the end of study unless the trial is stopped for some reason. A child may be excluded from the trial for the purposes of vaccination and/or clinical sampling or if ongoing participation is considered to be against their best interests or if a contraindication to vaccination and/or to the obtaining of clinical samples is identified. Such decisions will be made by the PI in discussion with other members of the clinical trial team and/or the sponsor. A child excluded in this way would be replaced and hence this would not alter the target sample size of 750. If not enough children from the previous cohorts are available, we will aim to recruit appropriate age groups from community health centres. We expect this to be an exception rather than the rule.

***Eligibility of participants***

Participants must meet all of the inclusion criteria and none of the exclusion criteria to be eligible to participate in the trial. A representative sample of children will be recruited from the target population.

Inclusion criteria:

Any child fitting the required age cohorts who has a documented record of having received a primary dose of the Institut Pasteur YF 17D vaccine between 9 and 12 months of age. Documented evidence can be either a previous record of YF vaccination with dates in our own trial registers if the child was a previous participant or has documented evidence on its Infant Welfare Card.

Exclusion criteria:

- Any child with a height/length for weight z-score of -3 or below.
- Any child known to be immunocompromised including any child with known vertical exposure to HIV infection.
- Any child with a history of serious adverse event or other contraindication to previous yellow fever vaccination.
- Participants who have an acute illness including abnormal vital signs or a fever of ≥ 37.5°C will not be vaccinated on the day but may be invited back for re-screening when they have recovered.
- Any child receiving immunosuppressive therapy.

***Concomitant medications***

Other than immunosuppressive drugs, no concomitant medication is excluded but will be noted in clinical files.

***Withdrawal of participants***

A study participant will be discontinued from participation in the study if:

- Any clinical significant adverse event (AE), intercurrent illness, or other medical condition or situation occurs such that continued participation in the study would not be in the best interest of the participant.
- Development of any exclusion criteria.

Participants are free to withdraw from the study at any time without giving a reason.

### Study procedures and evaluations

For an overview, see the below Schedule of Events ( Table 2).

**Table 2.** Schedule of Events.

Visit #	V1	Safety follow up visits	V1+7	V1+14	V2
Study Day	0	Visit 1 at home, visit 2&3 per phone unless concerns	7	14	28
Visit window (days)	-	Day 1–3 following vaccination	+1	+3	+14
Written informed consent	X				
Final eligibility confirmation	X				
History and physical examination	X				X
Vital signs & anthropometry	X		X	X	X
Blood sampling for YF serology and B cell memory	X				X
Study vaccine administration	X				
Solicited local and systemic AE data collection	X	X			
Unsolicited AE collection	X	X	X	X	X
T-cell responses (exploratory sub-study participants only)	X			X	
Transcriptome (RNA) (exploratory sub-study participants only)	X		X		
End of study visit					X

***Subject identification and sensitisation***

The databases of the previously conducted trials (summarized in Table 1 above) will be accessed and interrogated for potentially available children according to age groups at the time of recruitment to the current booster study. Potentially suitable children according to the specified age cohorts will be identified and contact details for their families confirmed via mobile phone calls. During such calls, it will be confirmed that the family is happy to be contacted for a further MRC study. If willingness to engage is confirmed, the broad details of the booster study are explained to the carer, and the carer and child will either be invited to a sensitization visit at the clinical trial site or via a home visit to share the informed consent document. The family is then given an appointment to attend the clinical trials site for Visit 1.

***Enrolment visit (Visit 1)***

At Visit 1, the informed consent procedures will be completed, and the eligibility criteria will be confirmed. Vital signs and anthropomorphic data will be collected. The pre-booster blood sample will be collected and the booster dose of the YF vaccine administered as per protocol by the study team. The child will be observed in the clinic for 30 minutes. At the end of 30 minutes it will be confirmed by a nurse to the study clinician that the child remains well prior to them leaving clinic.

***Follow-up***

Solicited adverse events (AE) will be collected up to and including Day 3 following the booster vaccination. On Day 1, a member of the field team will conduct a home visit to assess and record any local or systemic AE including the presence of fever. Any child with a local or systemic response of concern will be reviewed by a study clinician the same day or within 24 hours according to the nature of the complaint. On Day 2 and 3 following vaccination, a member of the field team will contact the family by telephone to follow up on any ongoing or new local or systemic reaction. If there are any additional concerns, a home visit or clinic visit will be arranged as judged necessary by the study clinician. After Day 3 and until the end of the study, unsolicited adverse events will be collected. Parents will be asked to contact the study team to report any potential adverse events. These will be reviewed accordingly, and safety data recorded.

Immunology subgroups:

30 children per age cohort will be included in the immunology sub-studies which require a further blood sample on either Day 7 (+/-1) or Day 14 (+/-3) following the booster vaccination. For this purpose, the child will attend the clinic for blood sampling (see Table 2).

***Final study visit***

On the final visit at Day 28 (+/-14), the child will be examined, and a further blood sample of 5–10 ml will be collected (5 ml for children under 5 years, up to 10 ml for older children).

***End of study***

The total duration of the clinical phase of the trial will be 12 months. This phase will end once up to 250 participants per age group have been enrolled and completed their D28 post booster vaccination visit. We will not substitute dropouts. The total duration of the trial is 18 months, and the trial will be considered as ended once the database has been cleaned and locked, the close out monitoring visit conducted and the end clinical study report submitted to MCA and to the IEC.

***Clinical evaluations***

During Visit 1 and Visit 2, clinical examination, vital signs, height and weight will be carried out and documented in the eCRF.

***Laboratory evaluations***

No routine clinical laboratory blood samples will be collected in this study. The research blood samples will be collected on V1 and V2 for YF serology and B cell memory responses for which PBMC will be isolated and stored. If, based on initial laboratory work, it is determined that a smaller number of PBMC will be required at this timepoint, serum will be collected alone.

30 children per age cohort will be included in the immunology sub-studies which require a further blood sample on either Day 7 (+/-1) or Day 14 (+/-3) following the booster vaccination. To this purpose, the child will attend the clinic for blood sampling.

At any timepoint, a maximum of 5 ml of blood will be collected from children younger than 5 years of age and up to 10 ml from older children.

Material Transfer agreements will be developed with any collaborating laboratories and valid certificates of accreditation will be provided for the external laboratory at the time of submitting the Material Transfer Agreement.

### Safety considerations

Yellow fever vaccines are generally exceptionally safe, and millions of doses have been given to adults and children worldwide. There have been rare reports of serious side-effects from the yellow fever vaccine. The rates for these severe ‘adverse events following immunization’ (AEFI), when the vaccine provokes an attack on the liver, the kidneys or on the nervous system are between 0 and 0.21 cases per 10 000 doses in regions where yellow fever is endemic, and from 0.09 to 0.4 cases per 10 000 doses in populations not exposed to the virus ^1^. The risk of AEFI is higher for people with primary or secondary immune deficiency who will therefore be excluded from this study. In addition, any child with a history of serious adverse event to the previous yellow fever vaccine would be excluded from the booster study.

***Adverse events***

An adverse event (AE) is any untoward medical occurrence in a study participant which does not necessarily have a causal relationship with either the vaccines administered or study participant. An AE therefore includes any unfavourable and unintended sign (including an abnormal laboratory finding), symptom, or disease occurring during the study period. Any AE which occurs after an immunization may be termed an AE following immunization (AEFI). For this trial, there will be active surveillance for local and/or systemic adverse events following immunization (AEFI) up to and including Day 3 following the booster vaccination ( Table 2A and Table 2B).

Established criteria will be used to record such events:

**Table 2A.** Local solicited adverse events.

Local administration site	Grade 0	Grade 1 Mild	Grade 2 Moderate	Grade 3 Severe	Grade 4 § Potentially Life Threatening
Pain	No pain	Pain causing no or minimal limitation to use of limb	Pain causing greater than minimal limitation to use of limb	Pain causing inability to perform usual social and functional activities	Pain causing inability to perform basic self-care function or hospitalization indicated
Redness/Erythema ‡ (size as well as grade will be collected)	No redness/ erythema	≤ 2.5cm in diameter	> 2.5cm in diameter with < 50% of the surface area of the extremity segment involved (e.g. lower arm or thigh)	≥ 50% of the surface area of the extremity segment involved (e.g. lower arm or thigh) OR ulceration OR secondary infection OR phlebitis OR sterile abscess OR drainage	Potentially life- threatening consequences (e.g. abscess, exfoliative dermatitis, necrosis involving dermis or deeper tissues)
Swelling/induration ‡ (size as well as grade will be collected)	No swelling/ induration	≤ 2.5cm in diameter	> 2.5cm in diameter with < 50% of the surface area of the extremity segment involved (e.g. lower arm or thigh)	≥ 50% of the surface area of the extremity segment involved (e.g. lower arm or thigh) OR ulceration OR secondary infection OR phlebitis OR sterile abscess OR drainage	Potentially life- threatening consequences (e.g. abscess, exfoliative dermatitis, necrosis involving dermis or deeper tissues)

§ any AE resulting in death will be defined as grade 5 severity. ‡ grading based on the greatest single diameter or measured surface area. Based on the National Institute of Health, Division of AIDS (DAIDS) Table for Grading the Severity of Adult and Pediatric Adverse Events – Corrected Version 2.1 July 2017.

**Table 2B.** Systemic solicited adverse events.

Systemic	Grade 0	Grade 1 Mild	Grade 2 Moderate	Grade 3 Severe	Grade 4 § Potentially Life Threatening
Acute allergic reactions	No acute allergic reaction	Localized urticaria (wheals) with no intervention indicated	Localized urticaria (wheals) with intervention indicated OR mild angioedema with no intervention indicated	Generalized urticaria OR angioedema with intervention indicated OR symptoms of mild bronchospasm (wheeze)	Anaphylaxis OR life-threatening bronchospasm (wheeze) or laryngeal oedema (stridor)
Axillary Temperature	< 37.5°C	37.5 to 38.4°C	38.5 – 38.9°C	39.0 – 40.0°C	> 40°C
Vomiting	No vomiting	Transient or intermittent AND no or minimal interference with oral intake	Frequent episodes with no or mild dehydration – oral rehydration solution indicated	Persistent vomiting resulting in orthostatic hypotension OR aggressive rehydration indicated (e.g. intravenous fluids)	Life threatening consequences (e.g. hypotensive shock)
Diarrhoea	No diarrhoea	Liquid stools (less formed than usual) but usual number of stools	Liquid stools with increased number of stools OR mild dehydration	Liquid stool with moderate dehydration	Life threatening consequences (e.g. liquid stool resulting in severe dehydration, hypotensive shock)
Irritability	No irritability	Crying more than normal/irritability but no or minimal interference with usual social and functional activities	Crying more than normal/irritability causing greater than minimal interference with usual social and functional activities	Crying more than normal/irritability preventing usual social and functional activities	Requiring hospitalization due to irritability
Drowsiness	No drowsiness	Sleeping more than normal/drowsiness but no or minimal interference with usual social and functional activities	Sleeping more than normal/drowsiness causing greater than minimal interference with usual social and functional activities	Sleeping more than normal/drowsiness preventing usual social and functional activities	Requiring hospitalization due to drowsiness
Appetite	Eating/ feeding normally	Eating/feeding less than normal but no or minimal interference with usual social and functional activities	Eating/feeding less than normal with greater than minimal interference with usual social and functional activities	Eating/feeding less than normal preventing usual social and functional activities	Requiring hospitalization due to not eating/feeding.
Rash at a site other than product administration site	No rash	Localized rash at a site other than the product administration site.	Diffuse rash OR target lesions	Diffuse rash AND vesicles or limited number of bullae or superficial ulceration of mucous membranes at one site	Extensive of generalized bullous lesions OR ulceration of mucous membranes involving two or more distinct mucosal sites OR Stevens-Johnson syndrome OR toxic epidermal necrolysis.

§ any AE resulting in death will be defined as grade 5 severity. Modified from the National Institute of Health, Division of AIDS Table for grading the severity of adult and pediatric AE – Version 2.1 July 2017; US Department of Health and Human Services, Food and Drug Administration, Guidance for Industry, Toxicity Grading Scale for Healthy Adult and Adolescent Volunteers Enrolled in Preventative Vaccine Clinical Trials, Sep 2007.

***Serious adverse events (SAEs)***

An AE will be considered to be serious (i.e. SAE) if:

- It results in death.
- Is life-threatening (an event where the participant was at risk of death at the time of the event).
- Requires in patient hospitalization or prolongations of existing hospitalization.
- Results in persistent or significant disability.

The start date for an SAE is defined as the day the relevant SAE criteria were met, not the day an AE which subsequently developed into an SAE began. The end date for an SAE is the day the final AE resolves, NOT the day the criteria rendering the AE serious resolves, i.e. if a participant is admitted with dehydration secondary to gastroenteritis, the SAE only ends when the diarrhoea resolves even if the infant has been discharged sometime before this. ‘Death’ and ‘Persistent or significant disability/incapacity’ will be defined as ongoing and will be closed for the purposes of further reporting once relevant clinical details have been captured (e.g. cause of death) and, in the case of disability/incapacity, if the condition is considered stable in the short term. 

***Reporting procedures***

SAEs will be reported by the PI to the sponsor and the local safety monitor within 24 hours of the investigator team becoming aware. The report should be submitted on the designated SAE reporting form for the trial. The reports will be submitted by e-mail or via the online reporting form-depending on MRCG SOPs. Follow-up reports will subsequently be submitted as new information becomes available until the SAE is closed or defined as ongoing.

Deaths occurring in the trial, irrespective of whether judged to be related or expected will be reported by the investigator by letter to the Gambia Government/MRCG joint ethics committee at their next meeting. SAEs that are judged to be related to investigational products and unexpected (SUSAR) will be reported by letter by the PI to The Gambia Government/MRCG Joint ethics committee within 24 hours.

SAEs that are judged to be related to investigational products but not unexpected will be reported by letter by the PI to The Gambia Government/MRCG Joint ethics committee within 15 calendar days or within 7 calendar days if the event is fatal or life threatening.

All SAEs will be reported by the PI to the Gambia Government/MRCG joint ethics committee in the annual report.

All deaths and all SAEs that are judged to be related to investigational products will be reported to the Republic of the Gambia MCA by the sponsor according to the latest requirements of the agency.

If new information becomes available that may alter the safety or conduct of the trial the investigator will inform the sponsor, the Local Safety Monitor, The Gambia Government/MRCG Joint Ethics Committee and The Gambia MCA in writing as soon as they become aware and generally within 5 calendar days.

***Safety oversight***

This trial uses an already licensed product that is in general use in The Gambia with an established safety record. The product has also been used in booster vaccinations. The trial is therefore considered to be relatively low risk. A DSMB will review safety data on a regular basis as per the DSMB charter. A local safety monitor is in place and will review local and systemic AEs and any SAEs, as will the trial management team on an ongoing basis.

### Statistical considerations

***Sample size considerations***

The primary endpoint of this study is the PRNT titres post booster vaccination according to intervals between primary YF vaccination and the booster dose. A sample size of 250 participants per cohort provides a precision of around +/- 5% over a range of plausible seroconversion rates associated with boosting at the three different timepoints ( Table 3).

**Table 3.** Precision around given estimates of seroconversion based on a sample size of 250 participants per group.

Seroconversion	95% confidence interval
95%	91.6 – 97.1
90%	85.7 – 93.1
80%	74.6 – 84.5
70%	64.1 – 75.3
60%	53.8 – 65.9
50%	43.9 – 56.2

The same sample size also provides over 80% power to detect a 10% difference in the seroconversion/seroreversion rates between two cohorts assuming a rate of at least 85% in one cohort, and over 90% power to detect a 15% difference in any two seroconversion/seroreversion rates.

Exploratory immunology sub-studies:

Our previous systems biology studies showed that significant differences in “omic” signatures over the first week of life and according to day of life were detectable in cohorts of infants as small as n=10 per group. We cannot currently confirm if such “omic” changes are informative to predict responses to YF vaccine in young children, but based on the data from Querec *et al.*, systems biology approaches were informative in adults in group sizes as small as n=15 ^25^. We are therefore confident that a group size of 30 per sub-group will provide us with sufficient datasets to not only explore if “omic” signatures in children are also predictive of humoral responses to the booster, but also to interrogate pathways and cellular populations involved in mediating correlates of protection against yellow fever.

***Data presentation and analysis***

Baseline data:

All baseline data including demographic and anthropological parameters will be tabulated by age cohort along with appropriate measures of spread. 

Immunogenicity data:

Descriptive evaluations including yellow fever PRNT GMT, seropositivity, seroreversion rates between 9 months and boost and seroconversion rates following boost (including the number of participants who seroconvert or who have a four-fold rise in antibodies concentrations) and relevant differences in these measures between age cohorts will be calculated and presented with appropriate measures of spread.

GMT data will be log _2_ transformed for analyses. Two-sided 95% confidence intervals (CI) for the log transformed means will be estimated assuming a Student’s t-distribution. These will be converted to the original scale by calculating 2 ^L ^: 2 ^U^, where L and U are the limits of the 95% CI of the log-transformed means.

GMT ratios and their 95% CIs will be calculated in the same manner. The log _2_ ratio is the mean difference of the log _2_ transformed data with a two-sided 95% CI estimated via a two-sample t-test and the interval is reverse transformed as above to obtain the 95% CI on the original scale. If, after log transformation, the normality assumption is not tenable, 95% CIs will be estimated using bootstrapping. Titre distributions by age cohort pre-boost and post-boost will be illustrated using reverse cumulative distribution (RCD) plots.

Safety data:

Summary statistics including appropriate measures of spread will be calculated for all safety data including solicited local and systemic AEs and unsolicited AEs. Safety events including AEs and SAEs will additionally be provided.

### Planned interim analyses

Safety endpoints will be reviewed by the DSMB at timepoints specified in the DSMB charter. No additional interim analyses are planned. No interim analyses of immunogenicity data are planned.

### Statistical Analysis Plan

Full details of the final statistical analysis to be undertaken in the trial will be set out in the statistical analysis plan (SAP). A full SAP detailing all the analysis and management of missing data is not available yet. This will be provided before database lock. In case of amendments or decision to deviate from the SAP, updated versions of the SAP will be communicated as well to the MCA.

### Data handling and record keeping

***Data Management and Processing***

Full details regarding the handling of trial data will be described in the trial data management plan (DMP) which will be consistent with appropriate data management SOPs of the Unit and applicable regulatory requirements. All documents used during the trial will be version controlled and dated.

Source will be defined for all data to be collected during the trial in a source data designation log. Electronic CRF (eCRF) will act as the source for certain data. Other data will be transcribed from alternative source documents. These might include the infant welfare card held by the mother, clinical notes, reactogenicity record forms and the vital signs and anthropometry card. Certified photocopies of relevant source documents will be made for subsequent source data verification (SDV) purposes when appropriate.

Subject data will be collected or transcribed contemporaneously into the eCRF according to written CRF completion guidelines for the trial. The eCRF will be developed with the input of sponsor, investigator and data management personnel. Draft eCRFs will be piloted by the investigator site and will be reviewed to ensure all protocol required data for the assessment of the trial endpoints are collected and also that the data required to confirm protocol compliance are captured.

Data entered into the eCRF will include screening number, initial immunization date with YF vaccine, information on the visit number, the date of the visit and, when relevant, the time that particular activities were undertaken (e.g. to confirm the time interval between vaccination and the recording of the post-vaccination vital signs). The individual member of the clinical trial team entering data into the eCRF will be captured as part of the entry process using a unique identifier. Such information will allow the designation of clinical trial staff completing the eCRF to be confirmed within the database.

***Data security***

During this study, we will be abiding to the General Data Protection Regulation and to our data protection policy, which mandates that all staff using personal data must ensure that they hold such data securely and that it is not disclosed to any unauthorised third party in any way, including by accident. Data breaches will be reported to the data protection officer immediately, to the IEC, and to the MCA. Investigations will be made to identify the root cause of the breach and addressed appropriately.

***Source documents and access to source data***

A subject folder will be maintained for all children and will contain the consent documentation, and the source documents including the clinical notes written on all participants in the trial.

The Principal Investigators will maintain appropriate medical and research records for this study in compliance with the principles of good clinical practice and regulatory and institutional requirements for the protection of confidentiality of participants. The study team members will have access to records.

The authorised representatives of the sponsor, the ethics committee(s) or regulatory bodies may inspect all documents and records required to be maintained by the investigator, including but not limited to, medical records (office, clinic, or hospital) for the participants in this study. The clinical study site will permit access to such records.

***Protocol Deviations***

A protocol deviation (PD) is any noncompliance with the clinical trial protocol, good clinical practice (GCP), or other applicable regulatory requirements. The noncompliance may be either on the part of the participant or the investigator, including the study team members, and may result in significant added risk to the study participant. As a result of a deviation, corrective actions will be developed and implemented promptly.

If a deviation from, or a change of, the protocol is implemented to eliminate an immediate hazard(s) to the trial participant without prior ethics approval, the PI or designee will submit the implemented deviation or change, the reasons for it, and, if appropriate, the proposed protocol amendment(s) as soon as possible to the sponsor for agreement and the relevant independent ethics committee (IEC) and MCA for review and approval before implementation.

The PI or designee will document and explain any deviation from the approved protocol on the CRF, where appropriate, and record and explain any deviation in a protocol deviation form that will be maintained as an essential document. All protocol deviation will be reported to IEC and to the MCA.

### Study monitoring

Study monitoring will be undertaken to verify that the rights and well-being of the children enrolled in the trial are protected, that the data reported are accurate, complete and verifiable from source documents and that the trial is conducted in compliance with the current trial protocol, with ICH-GCP guidelines, and with applicable regulatory requirements.

Full details of the monitoring to be undertaken prior to, during, and at the end of the trial will be documented in the trial monitoring plan which will be agreed and signed off by the sponsor prior to trial initiation. Monitoring will include a site initiation visit which will be undertaken prior to the first subject consent, on-site monitoring visits throughout the trial and a close-out visit. Remote monitoring of the trial database may also be undertaken.

### Ethical considerations

This study is conducted in accordance with the principles set forth in the ICH Harmonised Tripartite Guideline for Good Clinical Practice ^26^ and the Declaration of Helsinki in its current version ^27^, whichever affords the greater protection to the participants. The protocol was approved by the Gambia Government/MRC Joint Ethics Committee and the LSHTM Research Ethics Committee.

***General considerations on human subject protection***

The study staff will ensure that the participants’ anonymity is maintained. The participants will be identified only by a participant’s ID number on the CRF and any electronic database. All documents will be stored securely and only accessible by study staff and authorised personnel. The study will comply with the Data Protection Act, which requires data to be anonymised as soon as it is practical to do so.

***Rationale for participant selection***

The investigators aim to enrol children who have received a documented initial YF vaccine (in between the age of 9–11 months) while participating in previous trials conducted in The Gambia. Conducting this trial among children who had been participants in previous trials will enable the study to calculate more accurately the immunogenicity effect of a booster YF vaccine and assess the effect of time interval in between the first dose and the booster on the immunogenicity. On the other hand, there are additional data which were collected from these children during their participation in previous trials (such as anthropometric data), which will permit a better assessment of the immunogenicity of a booster dose, taking into account possible confounding factors. Finally, The Gambia is an endemic setting of YF, and shares many similarities with other endemic settings, which will facilitate future generalisability of the findings to the target population and drive a potential vaccine policy change in these settings.

***Evaluation of risks and benefits***

The risks to either infants, pre-school children or school age children taking part to this study are low, as the YF vaccines used in this trial are licensed, have proven to be safe, and these children have initially received a YF vaccine in previous trials and via routine EPI programs worldwide without raising major safety concerns. Also, the vaccines are currently used in the routine EPI schedule in The Gambia and as such trial participation per se is not associated with any increase in risk with this regard.

Nonetheless, the administration of any vaccine can be associated with local and systemic adverse reactions which can be severe or, exceptionally rarely, even life-threatening. The safety of participants will be monitored closely for 30 minutes following vaccination as standard practice by the investigator, local safety monitor and sponsor.

Blood sampling can cause some pain at the time and occasionally results in the development of a bruise but has no other risks. The volumes of blood to be taken, even in infants, are not of physiological significance.

Receiving a booster vaccine for Yellow Fever could be of significant benefit for these children living in an endemic setting for the disease and who, outside of this trial, are unlikely to receive this booster.

***Informed consent***

For those expressing an interest for their children’s participation, informed consent will not be undertaken on the same day as individual sensitization to ensure all caregivers have the chance to consider the information overnight, at least, prior to providing consent.

Written/thumb-printed informed consent will only be required from one caregiver, but in all cases, it will be confirmed that other caregivers are aware of the study and are supportive.

For children who are 6 years of age and above, oral assent will be obtained from them in addition to their caregiver’s written/thumb-printed consent. This oral assent will be documented in the clinical notes.

Information regarding the trial will initially be given to caregivers by a field worker or nurse, although all caregivers will also be given the chance to ask a study clinician any questions they may have regarding the trial prior to the assessment of understanding and consent documentation.

Given the low rate of English literacy and the vulnerability of the groups (children), the process of obtaining informed consent is of exceptional importance and will be a focus for trial monitoring activities. No remuneration for enrolling in the trial will be provided. Transport fares will be provided. All information required by ICH-GCP will be provided to potential mothers through the ICD. All ICD in use will be approved by the Gambia Government/MRCG Joint Ethics Committee. Should any new information which is considered to have potential implications for informed consent become available during the trial this will be made available to study participants and informed consent will be repeated using a further approved ICD. The provision of informed consent is an ongoing process and can be withdrawn at any time.

***Participant confidentiality***

All participant-identifiable information (names, addresses, contact details etc) will be held securely and will not be available to anybody other than those in the investigator team, external monitors or auditors working on behalf of the sponsor and, on request, and rightful individuals from the Gambia Government/MRCG Joint Ethics Committee and the National Regulatory Authority (The Medicines Board). Information on this will be included as part of the informed consent process. Paper records including identifiable source documents will be held securely in access-restricted filing cabinets. Electronic records will be on access-restricted servers in line with the applicable data security and data access policies.

***Future use of stored specimens***

Additional consent will be obtained for the storage and future use of any specimens remaining following the assessment of the specified endpoints for research of relevance to the people of The Gambia and related to vaccination, immunity and infection. Such additional consent is not required for enrolment in the main trial but will be obtained at the same time as consent to the overall trial. Following use of collected specimens for the primary, secondary and exploratory endpoints, the future use of any remaining stored specimens would require the approval of the Gambia Government/MRCG Joint Ethics committee.

### Publication policy

Trial results will be published in high-impact peer reviewed open access scientific journals as soon as they are available and will also be disseminated through presentations at scientific conferences and other academic meetings.

Results will be made available to research participants, their families, and to the participating communities at large through their presentation at community open days held at the clinical trial site.

No information on individual participants will be presented either in publications or other presentations and individual participants will not have access to their own results. This will be specified as part of the informed consent process.

### Roles and responsibilities

Chief Investigator: Prof Beate Kampmann, Theme Leader Vaccines & Immunity, MRC Unit The Gambia at the LSHTM

Principal Investigator: Dr Ebrima K Kanteh, Clinical Trials Coordinator, MRC Unit The Gambia at the LSHTM

Co-Investigator: Dr Ed Clarke, MRC Unit The Gambia at the LSHTM

Sub-Investigator: Dr Nabou Leigh, MRC Unit The Gambia at the LSHTM

Sponsor Representative: Elizabeth Batchilly Stanley, MRC Unit The Gambia at the LSHTM

Trial Monitor: Clinical Trials Unit, MRC Unit The Gambia at the LSHTM

Local Safety Monitor: Dr Uduak Okomo

Statistician: Dr David Jeffries, Head of Statistics, MRC Unit The Gambia at the LSHTM

DSMB Chair: Dr Suzanne Anderson, Imperial College London

Ethics Committees: Gambia Government/MRC Joint Ethics Committee, The Gambia, and LSHTM Research Ethics Committee, United Kingdom

National Regulatory Agency: The Medicines Control Agency, Serrekunda, The Gambia

## Discussion and conclusion

The published literature to-date, including data from our own studies, has indicated that yellow fever antibody titres potentially wane rapidly following primary immunisation as part of the routine immunisation programme. There is a paucity of data on the titres of neutralising antibody in children of different ages post primary YF vaccination, and in particular, no data on the immunological response to a YF booster at different ages in the paediatric population.

We therefore designed and conducted this trial to investigate the titre of neutralising antibody and response to a YF booster in three cohorts of children of different ages who all received primary YF vaccination at 9–10 months of age.

The WHO EPI schedule currently recommends a yellow fever vaccine at 9 months of age. The immunogenicity data from this trial may help to elucidate whether individuals vaccinated at 9 months require a booster dose of the vaccine to ensure long-term protection. The data generated by this trial will therefore be of critical public health importance and is expected to directly impact recommendations by the WHO Strategic Advisory Group of Experts (SAGE) working group on YF and thus inform YF vaccine scheduling in endemic countries ^11^. The YF SAGE working group leadership actively encouraged this trial and both the chief investigator (Prof. Kampmann) and co-investigator (Dr Clarke) are active members of this working group and can therefore guarantee the timely availability of the results. These results may lead to a revised SAGE recommendation, with policy change at a global level with particular relevance to children living in YF-endemic countries, or could provide reassurance for the current policy.

Further, if deemed necessary, this trial will also allow help to determine the optimal timing for a booster by delivering immunogenicity data from cohorts of different ages. However, the timing of a booster would also be guided by logistical, financial and health system constraints. An early booster, together with the measles booster (MCV2) at 15 to 18 months, would have significant logistic advantages in terms of achieving population coverage given existing drives to increase MCV2 coverage and the comparative ease of achieving coverage in this age group ^28^. However, the potential for high pre-existing neutralizing antibodies to limit the booster response in children has not been determined and remains of concern, given that studies of booster doses in adults have shown interference with pre-existing YF antibody ^18^. Potential interference between MCV2 and the YF booster could also impact long-term protection against YF ^7, 8^. Whether later administration of a booster may overcome these disadvantages remains to be established. Achieving coverage after the second year of life in most settings across sub-Saharan Africa would likely have significant logistical and cost implications, whether undertaken through routine immunisation services or through a campaign.

Finally, the systems biology data generated through this trial will provide novel insights to characterise the humoral and cell-mediated immune response to YF vaccination in children.

The trial has completed recruitment and follow-up and sample analysis is currently ongoing. We expect to publish the results from the main trial in late 2024, with findings from the exploratory sub-studies following soon thereafter.

## Ethics and consent

This study is conducted in accordance with the principles set forth in the ICH Harmonised Tripartite Guideline for Good Clinical Practice ^26^ and the Declaration of Helsinki in its current version ^27^, whichever affords the greater protection to the participants. The protocol was approved by the Gambia Government/MRC Joint Ethics Committee (approval number LEO26467, approval date 31 Dec 2021) and the LSHTM Research Ethics Committee (approval number LEO26467, approval date 11 Jan 2022).

For those expressing an interest for their children’s participation, informed consent will not be undertaken on the same day as individual sensitization to ensure all caregivers have the chance to consider the information overnight, at least, prior to providing consent.

Written/thumb-printed informed consent will only be required from one caregiver, but in all cases, it will be confirmed that other caregivers are aware of the study and are supportive.

For children who are 6 years of age and above, oral assent will be obtained from them in addition to their caregiver’s written/thumb-printed consent. This oral assent will be documented in the clinical notes.

Information regarding the trial will initially be given to caregivers by a field worker or nurse, although all caregivers will also be given the chance to ask a study clinician any questions they may have regarding the trial prior to the assessment of understanding and consent documentation.

Given the low rate of English literacy and the vulnerability of the groups (children), the process of obtaining informed consent is of exceptional importance and will be a focus for trial monitoring activities. No remuneration for enrolling in the trial will be provided. Transport fares will be provided. All information required by ICH-GCP will be provided to potential mothers through the ICD. All ICD in use will be approved by the Gambia Government/MRCG Joint Ethics Committee. Should any new information which is considered to have potential implications for informed consent become available during the trial this will be made available to study participants and informed consent will be repeated using a further approved ICD. The provision of informed consent is an ongoing process and can be withdrawn at any time.

## Data Availability

No data are associated with this article. Data sharing will be in accordance with LSHTM data management policy ^29^. Zenodo: Booster Vaccination against Yellow Fever in Gambian children-(BoVY) -a Phase 3 clinical trial to establish safety and immunogenicity of repeated YF vaccination in healthy Gambian children of different ages. https://doi.org/10.5281/zenodo.13369416 ^30^. This project contains the following extended data: Data Safety Monitoring Board/Independent Data Monitoring Committee Charter, available from) IDMC Charter BoVY. Data are available under the terms of the Creative Commons Attribution 4.0 International license (CC-BY 4.0). Zenodo: SPIRIT checklist for ‘Yellow Fever vaccine booster trial to establish safety and immunogenicity of repeated YF vaccination in healthy Gambian children of different ages: a phase 3 clinical trial protocol’, available from: https://doi.org/10.5281/zenodo.13369447 ^31^. Data are available under the terms of the Creative Commons Attribution 4.0 International license (CC-BY 4.0).
