# Supplementary material for: Booster Vaccination against Yellow Fever in Gambian children-(BoVY) -a Phase 3 clinical trial to establish safety and immunogenicity of repeated YF vaccination in healthy Gambian children of different ages
Source: Wellcome Open Res. 2024 Dec 24;9:733. [Version 1] doi: 10.12688/wellcomeopenres.23138.1 (PMC11811606; doi:10.12688/wellcomeopenres.23138.1)
Supplement: Peer review history

# Reviewer report: Reviewer response for version 1

**DOI:** 10.21956/wellcomeopenres.25478.r117591

**Published:** 2025-02-10

**Authors:** Aitana Juan-Giner

**Affiliations:** 1 Universitat Ramon Llull, Barcelona, Spain

The manuscript reports the study protocol of a safety and immunogenicity study to assess booster dose of yellow fever vaccine in a paediatric population of three different ages. The study should have been conducted in 2022. This study covers a knowledge gap and will provide important data to inform the revision of WHO recommendations on YF booster vaccination in children.

**The manuscript is overall very clear. The following suggestions could add clarity to the manuscript: **

- Please check the use of the spelling PRN vs PRNT.
- The authors state that consent for sample storage was obtained in the initial study. I recommend that the authors explain how the right to withdraw and continue providing consent to re-use previously collected samples and data was respected.
- The blood volume to be collected is specified to be between 5 to 10 ml depending on age. Would be good to include a rational of this difference (i.e. what would be done with the extra 5ml?)
- Laboratory analyses in the initial study were conducted at the Robert Koch Institute. In this study it is indicated that analyses will be conducted at Institute Pasteur and Robert Kock. I recommend including a rationale for the use of two laboratories and measures put in place to ensure comparability of results, as PRNT assay is not standardized. 
- The study will include a representative sample of three previous cohorts. Would be good to include how participants will be selected to achieve a representative sample. 

Is the study design appropriate for the research question?

Yes

Is the rationale for, and objectives of, the study clearly described?

Yes

Are sufficient details of the methods provided to allow replication by others?

Yes

Are the datasets clearly presented in a useable and accessible format?

Not applicable

Reviewer Expertise:

Vaccinology

I confirm that I have read this submission and believe that I have an appropriate level of expertise to confirm that it is of an acceptable scientific standard.

# Reviewer report: Reviewer response for version 1

**DOI:** 10.21956/wellcomeopenres.25478.r116874

**Published:** 2025-01-20

**Authors:** Charlotte Martin

**Affiliations:** 1 Infectious Diseases, CHU Saint-Pierre, Brussels, Belgium; 2 Université Libre de Bruxelles (ULB), Brussels, Belgium

This article presents a research protocol designed to determine the best time to administer a booster of YF vaccine to children who had received a conventional YF vaccination at 9 months of age, always at the same time as a measles vaccination, and the rate of seroreversion in these children between the vaccination at 9 months and the age of 15 months, 4 years or 8 and a half years. There is also an immunological study on three sub-groups to explore more extensively the immunological response to the YF booster vaccine.

 The protocol will administer this YF vaccine booster to children vaccinated at 9 months of age and followed up as part of a study in The Gambia, so as to take advantage of the high-quality demographic and immunological data already available for these children. The design of this study, using children who had already been monitored in the first study, meant that it was not possible to carry out a double-blind study.

**Positive points of this protocol:**

- Immunological sub-studies, including the study of early innate immunity.
- Patients vaccinated with vaccines administered in real life at 9 months.
- No vaccines administered concomitantly with the booster, so no immunological interference.

**Negative points of the protocol:**

- Sampling at 28 days post-booster is a little early, and there is a risk of overestimating the rate of presence of neutralising antibodies, or it would have been good to complete with one more sampling at 3-6 months.
- No vaccines administered concomitantly with the booster, so difficult to implement a potential future booster at a logistically convenient time with one or more other vaccines already present in the vaccination programme.

**Comments on the manuscript:**

- **Intro: **Add to the sentence on the waning of vaccine protection that vaccine coverage is very low in some countries, so re-emerging YF is not only because of breakthrough infections.
- In the causes of reduced protection, there is, importantly, the immune immaturity before the age of two, which often requires repeated immune stimulation.
- **Last sentence in the intro:** The importance of memory B-cells but above all, given the short incubation period, of maintaining circulating neutralising antibodies thanks to long-lived plasma cells.
- **Rationale:** The first sentence is redundant with the intro. Please note that the change at the WHO was implemented in 2016.
- **Cellular immune response:** I would put the considerations on the altered effectiveness of a booster already in the Introduction Section.

Is the study design appropriate for the research question?

Partly

Is the rationale for, and objectives of, the study clearly described?

Yes

Are sufficient details of the methods provided to allow replication by others?

Yes

Are the datasets clearly presented in a useable and accessible format?

Not applicable

Reviewer Expertise:

Infectious Diseases and Vaccinology, with a PhD on the Impact of HIV-1 infection on the humoral response to YF vaccination.

I confirm that I have read this submission and believe that I have an appropriate level of expertise to confirm that it is of an acceptable scientific standard.
